# Quantitative microbial population study reveals geographical differences in bacterial symbionts of *Ixodes ricinus*

**DOI:** 10.1186/s40168-022-01276-1

**Published:** 2022-08-04

**Authors:** Aleksandra I. Krawczyk, Lisa Röttjers, Manoj Fonville, Katshuisa Takumi, Willem Takken, Karoline Faust, Hein Sprong

**Affiliations:** 1 Centre for Infectious Disease Control, National Institute for Public Health and the Environment, Antonie van Leeuwenhoeklaan 9, Bilthoven, 3720 MA the Netherlands; 2 Laboratory of Entomology, Wageningen University & Research, Wageningen, The Netherlands; 3 Department of Microbiology, Immunology and Transplantation, Laboratory of Molecular Bacteriology, Rega Institute, Leuven, Belgium

**Keywords:** Tick-borne disease, Spotted fever rickettsiosis, Transmission dynamics, Quantitative microbiome analysis, Low-biomass samples

## Abstract

**Background:**

*Ixodes ricinus* ticks vector pathogens that cause serious health concerns. Like in other arthropods, the microbiome may affect the tick’s biology, with consequences for pathogen transmission. Here, we explored the bacterial communities of *I. ricinus* across its developmental stages and six geographic locations by the 16S rRNA amplicon sequencing, combined with quantification of the bacterial load.

**Results:**

A wide range of bacterial loads was found. Accurate quantification of low microbial biomass samples permitted comparisons to high biomass samples, despite the presence of contaminating DNA. The bacterial communities of ticks were associated with geographical location rather than life stage, and differences in *Rickettsia* abundance determined this association.

Subsequently, we explored the geographical distribution of four vertically transmitted symbionts identified in the microbiome analysis. For that, we screened 16,555 nymphs from 19 forest sites for *R. helvetica*, *Rickettsiella* spp., *Midichloria mitochondrii*, and *Spiroplasma ixodetis*. Also, the infection rates and distributions of these symbionts were compared to the horizontally transmitted pathogens *Borrelia burgdorferi* sensu lato, *Anaplasma phagocytophilum*, and *Neoehrlichia mikurensis*.

The infection rates of all vertically transmitted symbionts differed between the study sites, and none of the symbionts was present in all tested ticks suggesting a facultative association with *I. ricinus*. The proportions in which symbionts occurred in populations of *I. ricinus* were highly variable, but geographically close study sites expressed similar proportions. These patterns were in contrast to what we observed for horizontally transmitted pathogens. Lastly, nearly 12% of tested nymphs were free of any targeted microorganisms, which is in line with the microbiome analyses.

**Conclusions:**

Our results show that the microbiome of *I. ricinus* is highly variable, but changes gradually and ticks originating from geographically close forest sites express similar bacterial communities. This suggests that geography-related factors affect the infection rates of vertically transmitted symbionts in *I. ricinus*. Since some symbionts, such as *R. helvetica* can cause disease in humans, we propose that public health investigations consider geographical differences in its infection rates.

**Supplementary Information:**

online version contains supplementary material available at 10.1186/s40168-022-01276-1.

## Introduction

In Europe, *Ixodes ricinus* transmits a plethora of pathogens to humans, posing severe health concerns [1–3]. Recently, there is an increasing interest in how the community of microorganisms inhabiting a tick, or the so-called microbiome, affects its fitness and vectorial capacity. In the long term, understanding such effects of these microorganisms can lead to the development of novel and sustainable ways to combat ticks and tick-borne diseases, as it has been demonstrated for other arthropods [4, 5]. The microbiome may influence tick-borne pathogen transmission dynamics, for instance, by inducing the host immune system or altering the integrity of the host gut layer [6]. Studies have shown that the gut microbiota of the black-legged tick, *I. scapularis*, modulates the integrity of the peritrophic matrix that separates the lumen from the digestive cells of the gut and subsequently affects the establishment of *Borrelia* pathogens [7].

Ticks carry complex microbial communities that are largely dominated by nonpathogenic microorganisms exhibiting higher taxonomic diversity than tick-borne pathogens (reviewed in [8, 9]). Nevertheless, Little is known about how microorganisms, other than pathogenic agents, are acquired and propagated in *I. ricinus* ticks. Previous studies on the microbiome of *I. ricinus* detected large bacterial diversities, an overall unstable microbial composition, and an extremely low bacterial load in the midgut [9–13]. The microbiome of *Ixodes* ticks has been shown to vary on a spatial scale and through the ontogeny [14–18]. Moreover, recent studies have shown temporal dynamics of the tick microbiome, which is probably, due to differences in the composition of the environmental microbiota affected by fluctuations in abiotic factors such as temperature [19]. Lastly, Since *I. ricinus* has a three-host life cycle, feeding once per life stage, each feeding is an opportunity for a tick to acquire microorganisms in addition to acquisition from the vegetation, where it spends most of its life [18, 20]. *Ixodes ricinus* needs 2 to 4 years to complete its life cycle and consists of four life stages: egg, larva, nymph, and adult. In forested areas, larvae feed predominantly on rodents, nymphs on rodents and birds, and female adults on ungulates, mostly deer [21, 22]. After each blood meal, a tick moults into the successive life stage.

In other blood-feeding arthropods such as bed bugs, lice, and tsetse flies, it is common to harbour obligate symbionts, which are required to support normal host development. For example, they provide B vitamins and cofactors not usually obtainable from a blood-based diet [23–26]. In ticks, such intimate interactions are exemplified by *Rhipicephalus turanicus* and *Ornithodoros moubata* harbouring a *Coxiella*-like symbiont and *Francisella*-like symbiont, respectively. Genomes of both bacteria were shown to encode major B vitamin synthesizing pathways [27–30].

Interestingly, there is no strong evidence for an obligatory relationship between bacteria and *Ixodes* ticks. For example, in *I. scapularis* (evolutionarily closely related to *I. ricinus*), *Rickettsia buchneri* was considered an obligate symbiont as its genome contains all the genes of de novo folate (vitamin B9) biosynthesis [31]. However, on many occasions, *I. scapularis* ticks without *R. buchneri* have been reported. In fact, the prevalence of this symbiont in tick populations varied between 46 and 82% depending on the studied location, suggesting a facultative — not required for host survival — relationship rather than an obligate one [32, 33].

All microorganisms carried by ticks can potentially and occasionally infect humans and animals during tick feeding. However, some of them rely their life cycles on vertical transmission, namely they are passed on by female ticks to their offspring. Others rely on horizontal transmission and are acquired and propagated by ticks during blood feeding on vertebrates.

Various bacterial microorganisms such as *R. helvetica*, *Spiroplasma ixodetis*, *Midichloria mitochondrii*, and *Rickettsiella* spp. have evolved symbiotic relationships with *I. ricinus* [14, 34]. These symbionts are predominantly transmitted vertically [35, 36]. Therefore, they are detectable in questing larvae, the first mobile life stage, which did not have a chance to feed. Questing is a tick foraging behaviour such as climbing up grass or other structure to increase the chances of coming in contact with a suitable mammal host.

Presumably, *R. helvetica*, *S. ixodetis*, *M. mitochondrii*, and *Rickettsiella* spp. are dependent on ticks for their survival in nature, impact their tick hosts, and some of them, e.g. *R. helvetica*, are potentially pathogenic to humans. However, since their roles in the biology and ecology of *I. ricinus* are not fully understood, it is unclear what factors determine their prevalences in tick populations.

Contrary to vertically transmitted symbionts, most recognized tick-borne microorganisms that are pathogenic to humans are predominantly horizontally transmitted and can be found only in nymphs, which had a chance to feed once. These include pathogens such as *Borrelia burgdorferi* sensu lato, *Anaplasma phagocytophilum*, and *Neoehrlichia mikurensis*. Their infection prevalences in *I. ricinus* are mainly determined by local vertebrate communities [22, 37]. Exceptionally, some human tick-borne pathogens are transmitted both horizontally and vertically (e.g. *B. miyamotoi* or a protozoan parasite, *Babesia divergens*). T﻿he vertically transmitted microorganisms investigated in this study are referred to as symbionts whereas the strictly horizontally transmitted microbes as (human) pathogens. We believe that this division is relevant in understanding tick ecology.

In this study, we aimed to elucidate the dynamics of the bacterial community of *I. ricinus* with emphasis on symbionts, which may potentially influence tick vectorial capacity of pathogens. For that, we first used the 16s rRNA amplicon sequencing to explore the microbiome of *I. ricinus* ticks from six distinct geographical locations and of all life stages. Studies investigating the complete microbiome of *Ixodes* ticks often do not take a total bacterial load into account [14, 17, 18]. This can lead to spurious conclusions on the tick microbiome as low bacterial biomass might have important biological implications that are overlooked. Since the quantification of bacterial density can help better understand of tick physiology, this study adopted a qPCR approach inspired by studies on quantification of the bacterial microbiome [38, 39].

The microbiome analysis presented here allowed us to gain insight into microbial communities of *I. ricinus* and form the hypothesis that the prevalence of some vertically transmitted tick symbionts is determined on a larger geographical scale than observed for horizontally transmitted pathogens. To test this hypothesis, we screened with the symbiont-targeted qPCR nearly 17,000 individual questing nymphs of *I. ricinus*, collected from 19 forest sites in the Netherlands. Lastly, we compared distribution patterns of vertically transmitted symbionts with horizontally transmitted pathogen communities, which were quantified in a previous study [22].

## Materials and methods

Four different datasets were utilized in this study. Therefore, for clarity, each dataset has been summarized in Table 1.

**Table 1:** All datasets used in this study

Dataset (name)	Ticks (*n*)	Life stages	Locations in the Netherlands^a^	Year of sampling	Molecular technique	Source
Microbiome	655 in 133 pools	Larvae, nymphs, adult females and males	AW, DK, ST, BU, HD, ZM (*n* = 6)	2016	16S rRNA sequencing	This study
Symbiont	16,555	Nymphs	AW, DK, ST, BU, HD, ZM, PD, SD, VH, VA, MH, HM, BB, PW, DW, EN, RB, VL, KB (*n* = 19)	2013–2014	qPCR targeting: *R. helvetica*, *S. ixodetis*, *M. mitochondrii*, *Rickettsiella* spp.	This study
Pathogen	13,967	Nymphs	AW, DK, ST, BU, HD, ZM, PD, SD, VH, VA, MH, HM, BB, PW, DW, EN, RB, VL, KB (*n* = 19)	2013–2014	qPCR targeting: *B. burgdorferi* s.l., *A. phagocytophilum*, *N. mikurensis*	[22]
Transmission mode	1130	Larvae, nymphs, adult females and males	AW, ST (*n* = 2)	2019	qPCR targeting: *R. helvetica*, *S. ixodetis*, *M. mitochondrii*, *Rickettsiella* spp., *B. burgdorferi* s.l., *A. phagocytophilum*, *N. mikurensis*	This study

^a^The abbreviations of locations are explained in Additional file 1: Table S1

### Microbiome dataset

#### Sample collection and preparation for microbiome analyses of *I. ricinus*

**Fig. 1:**
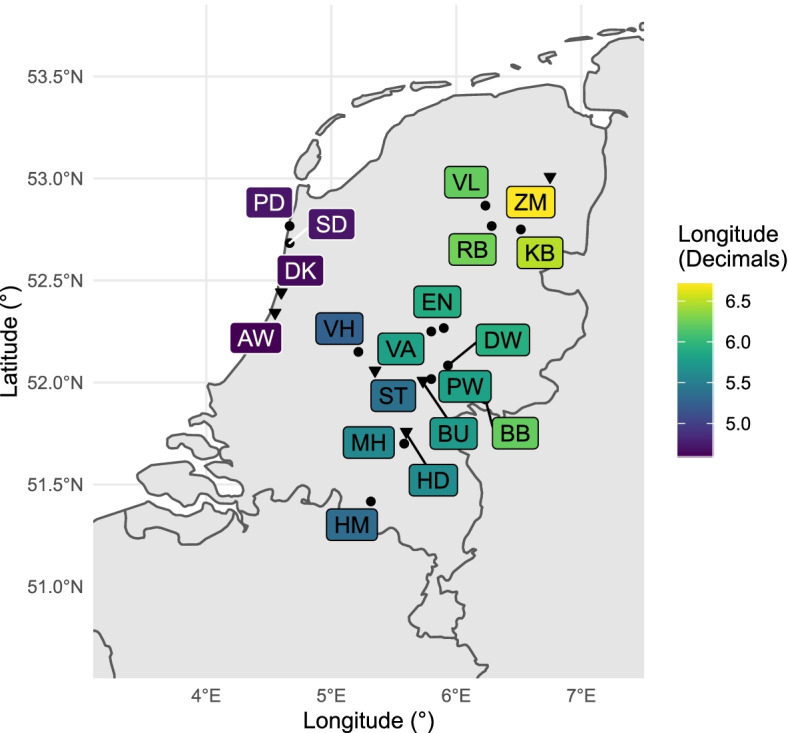
Sampling sites of *I. ricinus* in the Netherlands. Pooled and individual ticks from six forest sites (triangles) were used for a 16s rRNA amplicon 171 sequencing analysis. Individual nymphs from these and 13 (points) other forest sites were tested by 172 qPCR for the presence of tick symbionts. A box marks the sampling site by two letters, and a linear 173 colour gradient represents latitude-longitude. Full coordinates, habitat, vegetation cover, tick 174 densities, and a number of vertebrate species per locations are provided in Additional file 1: Table S1

We utilized pools of *I. ricinus* larvae, nymphs, and individual adult females and males from six locations across the Netherlands for microbiome profiling (Table 1; Fig. 1, triangles). These locations were selected based on pre-existing knowledge of *B. burgdorferi* s.l. prevalence, the density of ticks, vegetation profile, and vertebrate community obtained in a cross-sectional study [22]. The full names of the sites, their coordinates, and vegetation descriptions are provided in Additional file 1: Table S1.

Questing *I. ricinus* were collected in 2016 by blanket dragging. In total, 655 ticks were combined into pools by life stage and location. All ticks were washed three times in 70% ethanol, and DNA from 32 pools of 10 larvae, 18 pools of 5 nymphs, 18 pools of 10 nymphs, 34 individual female, and 31 individual male ticks was extracted using the QIAGEN DNeasy Blood & Tissue Kit according to the manufacturer’s protocol (Qiagen, Venlo, the Netherlands). A sampling scheme and sample metadata are provided in Additional file 1: Tables S2 and S3.

### Microbial profiling and taxonomic clustering

Illumina MiSeq V3-V4 region of 16S rRNA amplicon libraries were generated and sequenced by BaseClear (Leiden, the Netherlands). The description of the method has been published previously [40]. Besides tick samples, three negative controls were sequenced: two that went through the same processes as tick samples including crushing, extraction, and amplification and one included by the sequencing company.

Sequenced reads were imported to CLC Genomics Workbench 10.0.1 supplemented with CLC Microbial Genomics Module 3.6.1 (www.clcbio.com). Overlapping pairs of raw reads were merged into single longer reads and trimmed with a quality score limit of 0.05 and 2 ambiguous nucleotides. At this stage, primer sequences were trimmed. Subsequently, reads were fixed-length trimmed (~400 bp). To identify operational taxonomic units (OTUs), reads were clustered using the reference databases SILVA 16S version 128 with 97% identity as the clustering criterion; chimeras were removed.

### 16S rRNA quantification and total bacterial load

Total bacterial load in all samples was quantified, and proportions were multiplied by the load to convert relative into absolute abundances. Quantification of total bacterial DNA load was determined by 16S rRNA qPCR [38, 41, 42]. The details on a positive control, primers, protocol, and in silico analysis can be found in Additional file 2: Text 1. It should be noted that the primers were not developed specifically for tick-associated microorganisms, and that in this study, the 16S rRNA qPCR was used in addition to other methods.

The total bacterial load is a cost-effective and scalable solution for datasets of this size, since quantification methods through flow cytometry are not compatible with the sampling technique. Samples were normalized to control for arbitrary variation in sequencing depth, and the normalized abundances were scaled by the 16S rRNA qPCR values of each sample. For diversity analyses, samples were rarefied to the lowest sequencing depth.

### Microbiome analyses

All analyses were carried out in R 3.6.0 [43]. We used the R package *vegan* (version 2.5–6) for ordinations, diversity indices, and fitting of environmental vectors or factors onto ordinations [44]. We also computed silhouette scores from the Bray-Curtis dissimilarities and Sheldon evenness [45]. All principal coordinate analyses were carried out using Bray-Curtis dissimilarities, and *envfit* correlation to the principal components was corrected for multiple testing with the Benjamini-Hochberg correction. Additionally, we carried out PERMANOVA with the *adonis* function from R package *vegan* to assess whether tick life stage significantly affected community variation*.* We tested for multivariate spread through the *betadisper* function.

To test how well different factors explained clusters observed on the PCoA plots, we calculated the Bray-Curtis dissimilarities and evaluated cluster quality as the silhouette score with the factors as cluster labels. The silhouette score, bounded by −1 and 1, takes both cluster cohesion and cluster separation into account. We used k-means clustering of the log-transformed *Rickettsia* abundances to compute silhouette scores for *Rickettsia*.

To investigate correlations between OTUs and tick life stage, we fitted proportional odds models with OTU abundances scaled by the total bacterial load as the dependent variable and used tick life stage as independent variables. Details on models are provided in Additional file 2: Text S2. We compared these models with the likelihood ratio test, using an implementation of Nagelkerke’s pseudo-*R* ^2^ from the R package *rcompanion* (version 2.3.7) [46].

### Transmission mode dataset

A total of 1130 *I. ricinus* ticks of all developmental stages were collected in 2019 from two locations (ST and AW) and tested individually with qPCR for the presence of *S. ixodetis*, *R. helvetica*, *B. burgdorferi* s.l., *A. phagocytophilum*, and *N. mikurensis* for which primers and probes have been developed and published before [47–50]. In addition, ticks were tested for *Rickettsiella* spp., *M. mitochondrii* for which primers and probes were designed in this study. All targeted genes and qPCR protocol are provided in Additional file 2: Text S3. Subsequently, pathogens and symbionts were assigned the transmission mode based on their presence or absence in the larval stage indicating vertical or horizontal transmission, respectively.

### Dataset on vertically transmitted symbionts (symbiont dataset)

To determine the geographic distribution and prevalence of tick symbionts, we analysed a total of 16,555 ticks, which were collected in a previous cross-sectional study [21, 22]. Briefly, questing nymphs of *I. ricinus* were collected from 19 locations in forested areas in the Netherlands in 2013 and 2014 (Fig. 1, triangles and dots). Details on data collection were described previously [21, 22]. We tested questing individual nymphs of *I. ricinus* for the presence of *S. ixodetis*, *R. helvetica*, *Rickettsiella* spp., and *M. mitochondrii*.

### Dataset on horizontally transmitted pathogens (pathogen dataset)

In addition, we included in our analysis data on the prevalence and distribution of tick-borne pathogens (Table 1). This data was generated from the same tick collection, from 13,967 of 16,555 ticks, in the study of Takumi et al. [22]. The pathogens included *A. phagocytophilum*, *N. mikurensis*, *B. miyamotoi*, and three genospecies of *B. burgdorferi* s.l. as follows: *B. afzelii*, *B. garinii*, and *B. valaisiana*. Data on *B. garinii* and *B. valaisiana* were combined for further analysis as they are both considered bird-borne pathogens.

### Relative occurrence of vertically transmitted tick symbionts and horizontally transmitted pathogens

Based on the presence and absence of microorganisms detected with qPCR (symbiont and pathogen datasets), we assigned each tick a haplotype, an integer between 0 and 2^n-1^. Each integer corresponds to one of 2^n^ distinct outcomes for a series of n qPCR tests performed on the tick. Haplotype frequencies were arranged in a table where rows are the sampling sites and columns are the observed haplotypes. Each row was divided by the row sum. The column mean was subtracted from each column. Principal component analysis of the data table was performed by applying the singular value decomposition [51].

## Results

### Microbiome dataset

#### Microbial profiling and taxonomic clustering

A total of 131 of 133 processed samples generated 18,803,386 raw reads on Illumina MiSeq flow cell. Two samples failed at the amplification stage, probably due to low bacterial DNA load. A total of 6,013,524 sequences were assigned taxonomy. A total of 4978 unique OTUs were identified (Additional file 3: Table S4), and the top 10 most abundant taxa consisted of *Rickettsia*, *Rickettsiella*, *Midichloria*, *Pseudomonas*, *Halomonas*, *Rickettsiella*, *Mycobacterium*, *Shewanella*, *Methylobacterium*, and *Williamsia*.

**Fig. 2:**
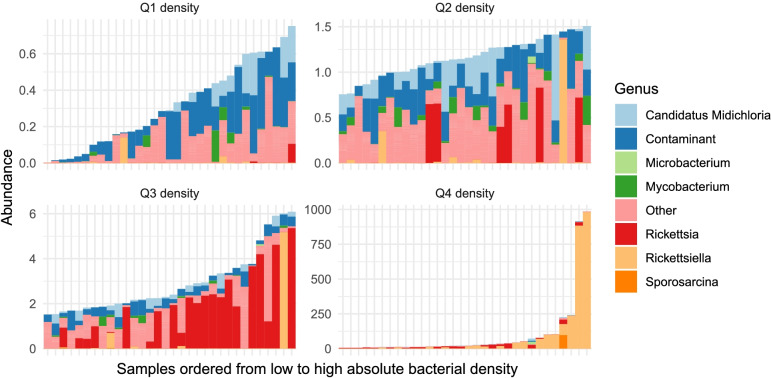
Abundances (relative abundances scaled by 16S rRNA content; Y-axis) of the ten most abundant bacterial taxa in individual and pooled *I. ricinus* samples (X-axis)

Three taxa, *Pseudomonas*, *Halomonas*, and *Shewanella*, were the most abundant in negative controls, indicating that these microorganisms are contamination from the processing or sequencing pipeline. Although we do not possess the reads from the internal negative control of the sequencing company, it has been confirmed that these three taxa were present in the company’s reagents. All counts belonging to these taxa were binned into a synthetic ‘contaminant’ taxon (Fig. 2). Several tick samples had extremely low biomass, and their community composition was similar to that of sequenced negative controls (Additional file 3: Fig. S1). These samples lacked tick-associated symbionts, and since the absence of a high-abundance symbiont can be considered a biological phenomenon, we chose to retain these samples, while the negative controls were excluded from further analysis. A full list of OTUs identified in the negative controls is provided in Additional file 3: Table S4.

Samples are separated by quartiles of total bacterial load (16S rRNA content in ng/μL). Bacteria present at a minimum threshold in sequenced control samples (blanks) were binned into the artificial ‘contaminant’ phylum. Taxa not in the top 10 were labelled ‘other’. A full list of detected bacterial taxa is provided in Additional file 3: Table S4.

Rarefaction curves indicated sufficient sequencing coverage for most samples, as demonstrated by most observed OTU accumulation curves having reached a plateau at 2000 reads (Additional file 3: Fig. S2). The low amount of total DNA and pooling strategy resulted in highly uneven sequencing depths Additional file 3: Fig. S3a). We assessed whether sequencing depth correlated to diversity since this would necessitate a rarefaction step. There was no apparent correlation between sequencing depth and diversity (Additional file 3: Fig. S4a), linear regression model *p* = 0.907, *R* ^2^ = −0.008), and we chose to avoid rarefaction before additional analyses were carried out.

### Abundance of tick-associated symbionts

Known members of the tick microbiome [34, 52, 53], the genera *Rickettsia*, *Midichloria*, and *Rickettsiella*, were among 10 of the most abundant taxa in the overall microbiome dataset accounting for 24.4%, 6.3%, and 17.1% of all reads, respectively (Fig. 2). Another tick-associated microorganism, *Spiroplasma*, was abundant but only in samples from two locations, ZM and AW, accounting for 0.3% of all reads [54]. The most abundant tick-borne pathogen in the dataset was *Neoehrlichia* (0.3%). Other pathogenic genera, including *Borrelia* and *Anaplasma*, constituted a small part of the overall tick microbiome accounting for 0.1% and 0.02% of all reads, respectively. Lastly, 0.4% of reads represented *Wolbachia* genus, probably due to endoparasitoid *Ixodiphagus hookeri* eggs in ticks [19, 55, 56].

### Effect of total bacterial load on the abundance of contaminants

Total bacterial load was weakly but significantly correlated with sequencing depth (Additional file 3: Table S5 and Fig. S5a, linear regression model *p* = 0.011, *R* ^2^ = 0.042). Moreover, 89.3% of samples had lower total bacterial load than the mean total bacterial load, meaning that the bacterial load was heterogeneous and skewed towards lower loads. Bacterial load was strongly correlated with the total number of uncorrected counts belonging to *Pseudomonas*, *Shewanella*, or *Halomonas* (Additional file 3: Fig. S5b, linear regression model *p* < 0.001, *R* ^2^ = 0.608). After scaling normalized sequencing counts by the total bacterial load, the bar plots of bacterial abundances demonstrate that the contaminants make up a large fraction of samples with low total bacterial loads (Fig. 2 and Additional file 3: Fig. S3b). In addition, the lower total bacterial load was correlated to the higher Shannon diversity (Additional file 3: Fig. S4b, linear regression model, *p* < 0.001, *R* ^2^ = 0.195).

### Effect of life stage and longitude on bacterial community variation

**Fig. 3:**
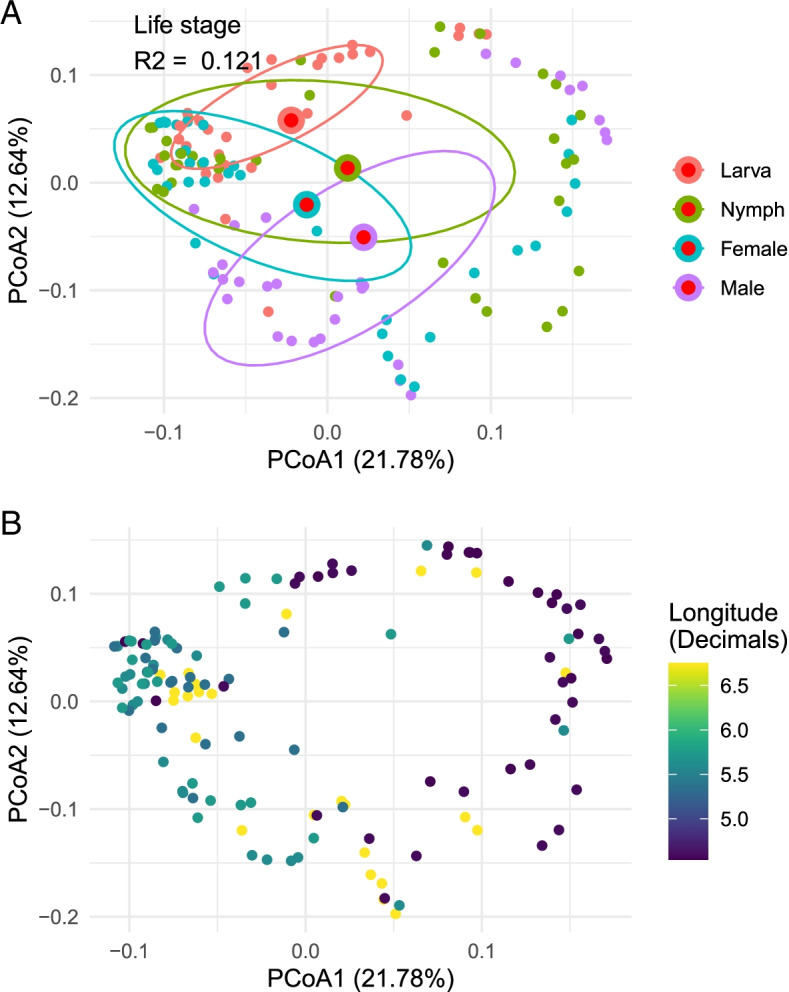
Bacterial community variation in Ixodes ricinus across life stages and longitude. **a** Principal Coordinate Analysis of Bray-Curtis dissimilarities overlaid with centroids of tick life stage. Data ellipses contain 50% of the samples belonging to the different life stages. **b** Principal Coordinate Analysis of Bray-Curtis dissimilarities, coloured by longitude

The marginal effect of life stage on bacterial community variation was significant, but the *R* ^2^ was small (*p* = 0.001, *R* ^2^ = 0.121, Additional file 4: Table S5). Our test for multivariate homogeneity of group dispersion was highly significant (*p* < 0.001, Additional file 4: Table S5). The silhouette score from the Bray-Curtis dissimilarities was 0.024, suggesting that samples are not tightly grouped by life stage (Fig. 3).

**Fig. 4:**
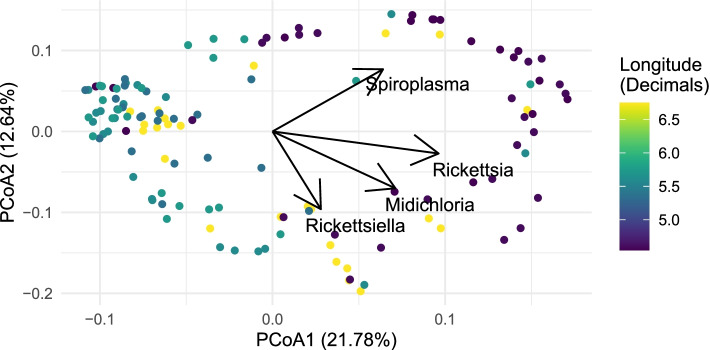
*Rickettsia* abundance covaries with community composition. Principal Coordinate Analysis of Bray-Curtis dissimilarities overlaid with envfit vectors for tick symbionts. Of these abundance vectors, only *Rickettsia* and *Rickettsiella* correlated significantly to the principal components (*p* = 0.001 for both). Nagelkerke’s pseudo-*R* ^2^ for different ordinal logistic regression models fitting scaled *Rickettsia* and *Rickettsiella* abundances is provided in Additional file 4: Table S6

Since the response of the bacterial community to tick life stage might not necessarily reflect the response of specific taxa, we investigated the link between *Rickettsia*, *Rickettsiella*, and life stage in more detail. Both *Rickettsia* and *Rickettsiella* had significant correlations to the axes (*p* = 0.001 for each), with a silhouette score of 0.122 for *Rickettsia*-based clusters suggesting that it could better explain community structure compared to tick life stage (Fig. 4). However, proportional odds models suggest that high *Rickettsia* and *Rickettsiella* abundances could not be explained with a model including only life stage as a factor (Additional file 4: Table S6). For both *Rickettsia* and *Rickettsiella*, the pseudo-*R* ^2^ was low at 0.051 and 0.009, respectively.

### Transmission mode dataset

*Rickettsia helvetica*, *S. ixodetis*, *Rickettsiella* spp., and *M. mitochondrii* were detected in all life stages (Additional file 4: Table S7), indicating vertical transmission. *Borrelia burgdorferi* s.l. (except one larva), *A. phagocytophilum*, and *N. mikurensis* were only detected in nymphal and adult stages (Additional file 4: Table S7). Our results corroborate the latter microorganisms enter a tick population via horizontal transmission while larvae feed on an infected host [57–59].

### Symbiont and pathogen datasets

#### Prevalence of tick symbionts and pathogens

**Fig. 5:**
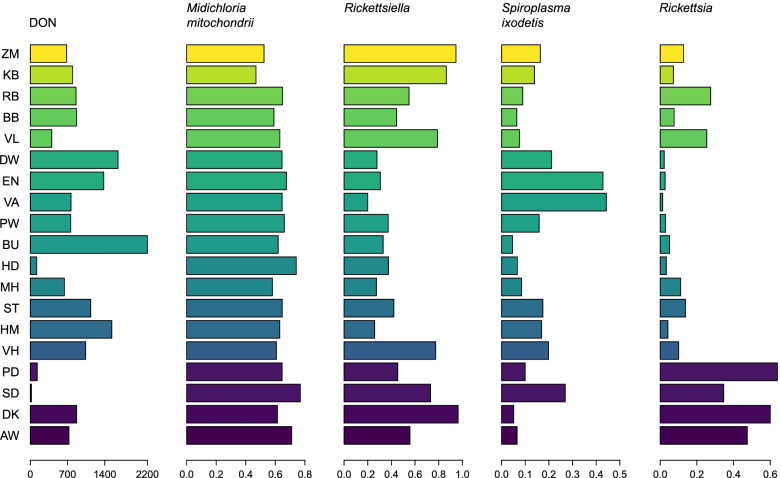
Local occurrence of vertically transmitted microorganisms. The leftmost column shows the density of nymphs (DON) per forest site (two-letter labels). The remaining columns show the prevalence of vertically transmitted tick symbionts. Locations are coloured by their longitude

The vertically transmitted symbionts were detected to a varying degree in all 19 forest sites (Fig. 5). Data on prevalence per symbiont per forest site as well as data generated in the previous study on three *B. burgdorferi* s.l. genospecies including *B. afzelii*, *B. garinii* and *B. valaisiana* (combined), and *A. phagocytophilum*, and *N. mikurensis* are provided in Additional file 4: Table S8. None of the nine microorganisms was detected in 12% of the tested nymphs (*n* = 1668), agreeing with the 16S rRNA amplicon sequencing results where many samples contained almost no microbiome (Additional file 3: Fig. S1).

### Relative occurrence of vertically transmitted symbionts

**Fig. 6:**
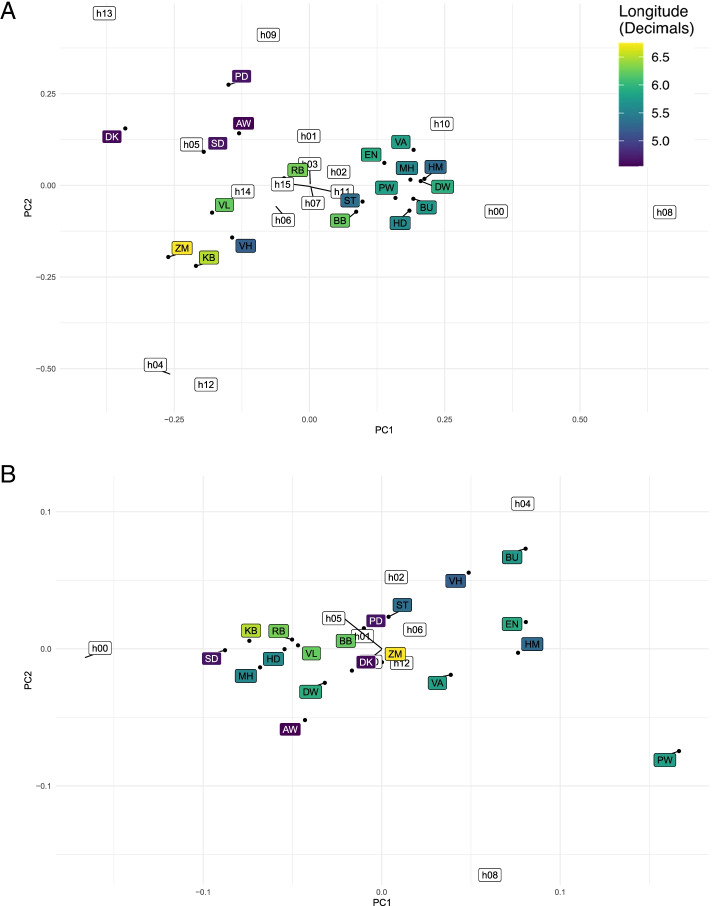
Relative occurrence of symbionts in *I. ricinus* nymphs from 19 forest sites coloured by their longitude. Percent of variance explained by each PC: **A** PC1 (56%) and PC2 (23%). **B** PC1 (69%) and PC2 (17%). **a** Relative occurrence of vertically transmitted symbionts such as *R. helvetica*, *M. mitochondrii*, *S. ixodetis*, and *Rickettsiella* spp. **b** Relative occurrence of horizontally transmitted pathogens such as *A. phagocytophilum*, *B. afzelii*, *B. garinii* and *B. valaisiana* (combined), and *N. mikurensis*. Tick populations from the forest sites situated close to each other (see Fig. 5) clearly share a similar composition of vertically transmitted symbionts but not horizontally transmitted pathogens. Transparent boxes show haplotype numbers, which correspond to symbiont combinations in individual ticks, for example in panel **a**, the forest sites from the centre of the Netherlands are dominated by nymphs infected only with *M. mitochondrii* (h08) or with none of the symbionts (h00). Please note the different scales of axes between the two figures. A full description of each haplotype is provided in Additional file 4: Table S9

We delineated the forest sites into three clusters according to specific combinations of infections in individual ticks (Figs. 1 and 6a). In cluster one, we found abundant questing nymphs in which all symbionts were absent (h00; Table S9 lists all the haplotypes) or in which only *M. mitochondrii* (h08 haplotype) was detected. In cluster two, questing nymphs were abundant, in which *Rickettsiella* spp. was present together with *M. mitochondrii* (h12) or without this species (h04). In cluster one, we found questing nymphs with *R. helvetica* and another symbiont: *M. mitochondrii*, *Rickettsiella* spp.*,* and *R. helvetica* (h13), *Rickettsiella* spp. and *R. helvetica* (h05), or *M. mitochondrii* and *R. helvetica* (h09). These three haplotypes (h13, h05, and h09) lacked *S. ixodetis*.

### Relative occurrence of horizontally transmitted pathogens

To contrast these results against pathogens that are transmitted horizontally, we performed the same analysis on *A. phagocytophilum*, *N. mikurensis*, *B. afzelii*, *B. garinii*, and *B. valaisiana* (combined). Contrary to the vertically transmitted symbionts, the forest sites did not cluster according to geographic proximity (Figs. 1 and 6b). The principal driver for differentiating the sampling sites was the absence of any of the horizontally transmitted pathogens in questing nymphal ticks (h00).

## Discussion

### Variable bacterial loads

Analysing ticks with next-generation sequencing comes with methodological challenges related to their overall low biomass. Samples with low total bacterial load suffer relatively more from contaminant DNA and cross-contamination than samples with high bacterial load [60]. Therefore, pooling tick samples can maximize microbial yield and provide a representative sample of microbial taxonomic diversity [14, 33, 61]. We used a similar approach in our study. Although we expected that the pooling would obfuscate the effects of bacterial biomass on community structure, 16S rRNA quantification demonstrated a surprisingly wide range of total bacterial loads across pools of ticks analysed in our experiment. While we could not determine the total bacterial load for a single tick directly, the negative correlation between established contaminants and total bacterial load suggests that our low microbial biomass pooled samples were more affected by contaminating DNA than high-biomass samples (Additional file 3: Fig. S5b). A similar negative correlation was found for Shannon diversity and total bacterial load, suggesting that diversity metrics are inflated for these low-biomass pools (Additional file 3: Fig. S4b), possibly due to higher sampling depth [62]. Neither the increase in contaminants nor the increase in diversity appeared to be a function of reduced sequencing depth, as the bacterial load was only weakly correlated to the sequencing depth (Additional File 3: Fig. S5a). However, by scaling microbial abundances through 16S qPCR quantification, the absolute abundances of the contaminants and rare species were reduced, while absolute abundances of high-density samples were reflective of the community. It should be noted that the quantification method used in this study might not be optimal to detect all tick bacteria which could lead to the underestimation of the tick bacterial loads. Ideally, the primers used in the quantification qPCR should be identical with those used for Illumina sequencing to optimally determine the absolute bacterial loads. Nevertheless, our results show that quantitative techniques for microbiome studies can facilitate identifying low-biomass samples in data sets with heterogeneous microbial load, even when used in combination with a pooling strategy commonly used to sequence low-biomass arthropod microbiomes.

### Limited microbiome

We observed similarities between sequenced negative controls and low-biomass samples. Many *I. ricinus* individuals appeared to harbour a limited microbiome, and whatever microbiome they did harbour could not be distinguished from negative controls based on Bray-Curtis dissimilarity (Additional file 3: Fig. S1). However, since this dissimilarity takes absolute counts into account, it emphasizes the low biomass of these samples rather than their composition. The genera found in these samples did appear to be distinct, with low-biomass samples containing genera unique to the tick microbiome such as *Midichloria*, *Rickettsia*, *Rickettsiella*, and *Spiroplasma* (Additional file 3: Fig. S6). Therefore, we chose to retain these samples since they may represent a condition of biological interest: the absence of any abundant tick symbiont. Yet, some samples had low relative abundance or were devoid of these genera, which are vertically transmitted symbionts previously associated with *I. ricinus* [34]*.* We analysed a similar tick population with symbiont targeted qPCR, to test this hypothesis since next-generation sequencing approaches cannot distinguish true absence from absence due to low sequencing depth. Interestingly, 12% of ~15,000 questing nymphs did not carry any of the four abovementioned vertically transmitted symbionts and any of five horizontally transmitted pathogens (*B. afzelii*, *B. garinii*, *B. valaisiana*, *A. phagocytophilum*, or *N. mikurensis*). In addition, with the microbiome analysis, we did not identify any other potential symbiont of *I. ricinus*.

Our results suggest that individual ticks can live devoid of bacterial symbionts. However, it remains unclear whether they can successfully feed or reproduce and, if not, what their role is in sustaining a tick population. Alternatively, ticks can harbour undetectable, by techniques used in this study, abundances/loads of bacterial symbionts. This could be happening when ticks are not actively metabolizing. Possibly, to maintain a symbiotic relationship, ticks downregulate the growth of intracellular symbionts during periods when symbiosis is the most expensive. For instance, in tsetse flies and algae, the load of symbionts has been observed to change through host development and environmental perturbations (such as changing humidity and light levels) [63, 64]. Lastly, *I. ricinus* might possess viral, eukaryotic, or perhaps archaea bacteria as obligatory symbionts.

We propose that *I. ricinus* as a species possess a highly variable microbiome, and no obligate bacterial symbiont is present in 100% of the population. These indications are somewhat unexpected given that ticks are arthropods feeding exclusively on vertebrate blood, a nutritionally restricted diet. In many hematophagous arthropods, including other tick species, obligate and maternally acquired bacterial symbionts are necessary for providing essential metabolites such as vitamin B that are deficient in vertebrate blood [27]. It is also possible that multiple symbionts can fulfil this and other crucial functions in *I. ricinus*, so that ticks may only need a single facultative symbiont from a pool of candidates. For instance, in aphids, *Serratia symbiotica* and *Hamiltonella defensa* confer similar benefits to their hosts as reviewed in Guo et al. [65]. Both symbionts have been shown to defend aphids against a parasitoid wasp [66] and improve host survival when subjected to heat shock [67]. Here, we observed that *S. ixodetis* occurred at the highest prevalence in ticks from the study sites with the lowest prevalence of *R. helvetica* (Fig. 5). Possibly, these two symbionts play similar biological function in *I. ricinus*, and bearing both of them is energetically costly to ticks.

When it comes to the lack of an obligate symbionts, similar observations have been made for *I. scapularis*. Previously, *R. buchneri* was considered an obligate symbiont of this tick species as its genome contains all the genes of de novo folate (vitamin B9) biosynthesis [31]. However, on many occasions, *I. scapularis* ticks without *R. buchneri* have been reported. The prevalence of this symbiont in tick populations varied between 46 and 82% depending on the location, suggesting a facultative over the obligate relationship [33, 68].

In addition, an in-depth study on *I. scapularis* suggested an unstable midgut microbiome [69], and a recent study by Guizzo et al. [13] showed an extremely low overall bacterial load in the *I. ricinus* midgut. These results are in line with an increasing body of evidence that many animals, including arthropods, are minimally or facultatively dependent on bacterial microbes or may not need a microbiome at all [70]. Thus, the observed variability of the tick microbiome likely represents transient associations with bacteria from the abiotic and biotic environment. In the recent review by Narasimhan et al. [71], it has been discussed that these transient microbial associations are under surveillance by tick innate immune responses. Subsequently, many microbes are cleared by effector molecules such as antibacterial peptides and potentially excreted due to the absence of cognate adhesins to engage with the tick gut [71].

### Microbiome of different tick life stages

We have observed small but statistically significant differences between microbiome compositions of distinct developmental stages of *I. ricinus*. The developmental stage on its own did not explain the clustering of tick microbiomes (Fig. 3A). Contrary to what we had expected, bacterial diversity seems to decrease along with tick development, illustrated with a significant difference between larvae and females. However, the opposite trend was observed from nymphs to adult males. Furthermore, the results suggest that during the off-host phases of ticks, fewer bacterial species enter a tick than has been speculated before [9]. Alternatively, the decreased bacterial diversity in females that we observed arose from the technical limitations of next-generation sequencing. It has been shown that abundant bacteria recruit more reads and potentially mask less abundant ones making them less likely to be detected [72, 73].

Previous studies have reported significant differences in *Ixodes* microbiome compositions of distinct life stages. However, they reported contradictory results regarding the dynamics of bacterial diversity. Zolnik et al. [16] documented increasing diversity, while Kwan et al. [17], Swei and Kwan [18], and Carpi et al. [14] showed decreasing diversity along with tick development. Therefore, the role of the life stage in shaping the tick microbiome is still unclear, and it is difficult to subtract this information from the data obtained solely with next-generation sequencing. Thus, in future studies, combining this method with other detection techniques is highly advisable.

### Microbiome and geographical location

In this study, the microbiomes of *I. ricinus* differed between distinct geographical locations. By scaling locations by their longitude, we could visualize that the change in the tick microbiomes occurred gradually rather than randomly (Fig. 3B). Thus, the bacterial communities of ticks originating from geographically distinct forest sites were significantly different and clustered apart. Previous studies also showed that the microbiome of *Ixodes* ticks varies by geographic location [14, 15]. These studies have given several probable explanations for differences in bacterial communities suggesting that they arose from the distinct habitats with a dissimilar availability of vertebrate hosts and consequently different environmental and animal host-associated bacteria [14]. Nevertheless, both differences in habitat and animal composition are locally determined, and the results in our study indicate that the factors driving differences in the microbiome are distributed over a broader geographical range.

### Distribution of tick symbionts

The observed clustering of the microbiomes was predominantly caused by varying tick symbiont communities. The strongest determinants were the abundance and prevalence of *Rickettsia* and *Rickettsiella* (Fig. 4). Therefore, based on these results, we hypothesized that the distribution and prevalence of *R. helvetica* (the most common *Rickettsia* species in *I. ricinus*) and *Rickettsiella* spp. are determined on a larger spatial scale than a single forest site. In addition to these symbionts, we studied *S. ixodetis* and *M. mitochondrii*, which were highly abundant in our microbiome dataset but less responsible for variations in bacterial communities of ticks. The prevalences of *R. helvetica*, *Rickettsiella* spp., *M. mitochondrii*, and *S. ixodetis* in questing nymphs were significantly different between the forest sites. Although all symbionts were present in all 19 studied locations and often at high prevalence, they occurred in varying proportions. The forest sites, which expressed similar proportions of symbionts, were also geographically close, consistent with what we observed in the microbiome dataset (Figs. 3B and 6A). We did not observe this pattern in horizontally transmitted pathogens such as *B. afzelii*, *B. garinii*, *A. phagocytophilum*, and *N. mikurensis*. The proportions in which they occurred were more random and varied between geographically close forest sites (Fig. 6B). This result was not surprising because horizontally transmitted pathogens have been shown to be determined mainly by local vertebrate communities [22, 37].

Nevertheless, mechanisms causing the heterogenicity in the prevalence of tick symbionts remain to be determined. Previous studies on host-symbiont interactions correlated variation in symbiont prevalence with environmental variables; a symbiont is more prevalent in the tick host population when it provides tolerance to a given biotic or abiotic stress and less prevalent when it is less beneficial for mitigating stress (and therefore, stress is a weaker selective force [34, 72, 73]. Therefore, (meta)populations of ticks from the western, northern, and central regions of the Netherlands may be exposed to different stresses. Abiotic stresses may arise from differences in soil type, temperature, and humidity related to coastal (west) and inland (centre and north) areas of the Netherlands. Biotic stresses such as the presence and abundance of tick parasites, for example parasitic wasps, might lead to varying prevalences of facultative defensive symbionts in tick populations as demonstrated for other species of arthropods [74]. Alternatively, the varying prevalences of a symbiont between distinct tick (meta)populations may arise from differences in transmission rate. The transmission rate could be potentially affected by a tick or symbiont genotypes or the compatibility of particular tick — symbiont genotype pairs [73]. This evolutionary divergence within *I. ricinus* and/or a symbiont species could be promoted by a geographical barrier and limited exchange of tick individuals between different parts of the country. However, this variation has not yet been studied and documented for tick — microbe symbiosis, and here, we did not look at the genetic diversity of neither *I. ricinus* nor symbionts.

The remaining question is whether the observed symbiont distribution is maintained in time. Although we did not include a temporal scale, a recent study by Lejal et al. [19] has shown that the *I. ricinus* microbiome may vary markedly throughout a year. Interestingly, this dynamic was explained by cuticle-associated bacteria rather than tick symbionts.

## Conclusions

*Ixodes ricinus* has a limited bacterial microbiome both in load and diversity and appears to lack an obligate symbiont. Nevertheless, given the relatively high prevalences of a variety of symbionts such as *S. ixodetis*, *R. helvetica*, *M. mitochondrii*, and *Rickettsiella* spp. in questing ticks, symbiotic bacteria appear to be intertwined with the biology of ticks. Varying symbiont functions and mechanisms underlying tick-symbiont interactions, which remain unidentified, may affect their distribution in tick populations. This, in turn, may have significant ramifications for generating the risk of diseases caused by *S. ixodetis* and *R. helvetica*. Here, we learned that *R. helvetica* displays characteristic geographical differences being highly prevalent in the coastal area vs inland. It is striking that such significant variation in prevalence can be observed already in a (small) country like the Netherlands. Whether this phenomenon also holds true for other more severe spotted fever group rickettsia’s from other tick species, like *R. conorii* and *R. rickettsii*, is uncharted territory and subject of future studies. Our findings imply that public health investigations and measures for etiological agents, such as risk assessments, implementation of diagnostic modalities, and preventive measures, should consider these geographical differences in the prevalence of *R. helvetica*.

## Supplementary Information

**Additional file 1: Table S1.** Metadata for all of the locations including their full names and coordinates as well as information on vegetation and tick density. **Table S2.** Number of samples collected from different tick life stages at different locations. **Table S3.** Location and tick life stage per sample.

**Additional file 2: Text S1.** Details on 16S rRNA quantification and total bacterial load. **Text S2.** Details on models investigating effect of life stage on bacterial community variation. **Text S3.** Primers and probes for the detection of *Rickettsiella* spp. and *M. mitochondrii*, target genes for the remaining symbionts and pathogens, and a qPCR protocol.

**Additional file 3: Table S4.** Read counts for different taxa, including taxonomy per sample. Negative control samples included. **Figure S1.** Ordination of community composition with uncorrected abundances. **Figure S2.** Rarefaction curve of samples coloured by quartile of total bacterial load. **Figure S3.** Abundances of bacterial phyla separated by quartiles of total bacterial load (16S rRNA content in ng/μL), unscaled (a) or scaled by total bacterial load (b). **Table S5. Figure S4.** Correlation of Shannon diversity of the unrarefied data to (a) sequencing depth and (b) total bacterial load. **Figure S5.** Correlation of total bacterial load to (a) sequencing depth and (b) uncorrected count of contaminant sequences. **Figure S6.** The top 10 genera of negative controls versus the 25% lowest biomass samples.

**Additional file 4: Table S5.** Results of PERMANOVA testing an effect of tick life stage on community variation, and the analysis of variance. **Table S6.** Results of models of stage explaining *Rickettsia* and *Rickettsiella* abundance. **Table S7.** Prevalence (%) of tick symbionts in larvae, nymphs and adults of *I. ricinus* collected in two locations (ST and AW) indicating primary transmission mode (vertical or horizontal). **Table S8.** Prevalence (%) of tick symbionts in nymphs of *I. ricinus* collected in 19 forest sites in the Netherlands. **Table S9.** A list of all haplotypes used in relative occurrence analyses.

## Data Availability

Demultiplexed raw sequence reads supporting the conclusions of this article were deposited into the Sequence Read Archive (SRA, PRJNA813158). The rest of the data generated or analysed during this study is included in this published article and its additional files.
