# Supplementary material for: Quantitative microbial population study reveals geographical differences in bacterial symbionts of Ixodes ricinus
Source: Microbiome. 2022 Aug 4;10:120. doi: 10.1186/s40168-022-01276-1 (PMC9351266; doi:10.1186/s40168-022-01276-1)
Supplement: Supplementary file 2 — Additional file 2: Text S1. Details on 16S rRNA quantification and total bacterial load. Text S2. Details on models investigating effect of life stage on bacterial community variation. Text S3. Primers and probes for the detection of Rickettsiella spp. and M. mitochondrii, target genes for the remaining symbionts and pathogens, and a qPCR protocol. [file 40168_2022_1276_MOESM2_ESM.docx]

## **Text S1**

**Details on 16S rRNA quantification and total bacterial load**

The bacterial load was quantified by quantitative PCR (StepOnePlus Real-Time PCR System, Thermo Fisher Scientific, the Netherlands) with universal primers and probe targeting the 16S rRNA gene, containing forward primer 16S-F1 (5′-CGA AAG CGT GGG GAG CAA A-3′), reverse primer 16S-R1 (5′-GTT CGT ACT CCC CAG GCG G-3′) and probe 16S-P1 (FAM-ATT AGA TAC CCT GGT AGT CCA-ZEN) (IDT, Leuven, Belgium). To optimize qPCR reproducibility and to allow comparisons of DNA concentrations reliably, we developed a standard curve by using a synthesized fragment of the 16S rRNA gene (gBlocks Gene Fragment, IDT, 5′-CGG TGC GAA AGC GTG GGG AGC AAA CAG GAT TAG ATA CCC TGG TAG TCC ACG CCG TAA ACG ATG TCT ACT AGC TGT TCG TGG TCT TGT ACT GTG AGT AGC GCA GCT AAC GCA CTA AGT AGA CCG CCT GGG GAG TAC GAA CGC AAG-3′). CT values were related to the standard curve ranging from 0.1 pg/µl to 1 ng/µl of bacterial DNA.

**Primers’ melting temperature**

As assesed with IDTdna (<https://www.idtdna.com/pages>), melting temperature of the forward and reverse primers were 65.9°C and 66.9°C, respectively.

**qPCR protocol**

PCR mix: 12,5 µl Diagnode universal Mastermix (Hotstart Taq NA polymerase, optimized reaction buffer, 4mM MgCl2 and dNTPs), 1 µl of each primer (10µM), 1 µl of the probe (5µM), 6,5 µl of DNA free water and 3 µl of template DNA.

Thermal cycling conditions: initial DNA denaturation step 2 min 50 degrees Celsius and 10 min 95 degrees Celsius, followed by 45 cycles of 15 sec 95 degrees Celsius, 1 minute 60 degrees Celsius.

**In silico primer performance**

The results of in silico analysis in the Silva database (<https://www.arb-silva.de/search/testprime/>) assessing the performance of the set of primers used in this study for the bacterial quantification approach (16S-F1 (5′-CGA AAG CGT GGG GAG CAA A -3′), reverse primer 16S-R1 (5′-GTT CGT ACT CCC CAG GCG G-3′):

0 Mismatch: Bacteria 0%

1 Mismatch: Bacteria (4.8%) and Archaea (0%)

2 Mismatches: Bacteria (58%) and Archaea (2.8%).

3 Mismatches: Bacteria (83.8%) and Archaea (35.9%)

## **Text S2**

**Details on models investigating effect of life stage on bacterial community variation.**

To investigate correlations between OTUs and tick life stage, we fitted proportional odds models with OTU abundances scaled by absolute bacterial density as the dependent variable and used tick life stage as independent variables. The model is defined as logit[P(Y_ik≤j)]= θ_j-x_i β_1 , where Yik is the OTU abundance that can fall in j categories, xi is a vector of life stage factors and β is the regression parameter for this factor. We used the clm function from the R package ordinal (version 2019.4-25) to fit these models (Christensen 2015). With the logit link function, coefficients can be interpreted as the effect that life stage factors have on the probability that a taxon reaches a certain level of abundance. We compared these models with the likelihood ratio test, using an implementation of Nagelkerke’s pseudo-R2 from the R package rcompanion (version 2.3.7) (Mangiafico and Mangiafico 2017).

Christensen RHB. Analysis of ordinal data with cumulative link models—estimation with the R-package ordinal. R-package version 2015:1–31.

Mangiafico S, Mangiafico MS. Package ‘rcompanion.’ Cran Repos 2017:1–71.

## **Text S3**

**Primers and probes for the detection of *Rickettsiella* spp. and *M. mitochondrii*, target genes for the remaining symbionts and pathogens, and a qPCR protocol.**

**Detection of symbionts**

*Rickettsiella* spp. qPCR
To detect Rickettsiella spp., two regions were targeted: a 196-bp fragment of the glucose-inhibited division gene using primers 5’-TGT AAT CCT TGA GTC TGA TCG T-3’, 5’-CAA ACC GAT ATG AAT TTT TCC GG-3’ and a probe 5’-ATTO520-TAG TTG GTG TGG TAA CGC AAA TGG GGT-BHQ2-3’, and a 76-bp fragment of the dihydrolipoamide succinyltransferase gene using primers 5’- GAT CAA CCC TCT CAA TCA GC-3’, 5’- GCC AAA TGG GTG TCA CTA T’ and a probe 5’-ATTO647- CAC CCG TCG CAG AAA AAA CTA AAC CTG-BHQ2-3’. Samples positive for at least one marker was considered positive for Rickettsiella spp.

*Midichloria mitochondrii* qPCR
To detect M. mitochondrii, one region was targeted: a 145-bp fragment of the gyrase subunit B gene using primers 5’- CTT GAG AGC AGA ACC ACC TA-3’, 5’- CAA GCT CTG CCG AAA TAT CTT-3’ and a probe 5’-ATTO424- GAG GGC GGA GTC AAA GAA TTT GTC CAC G-BHQ1-3’.

Targeted genes for remaining symbionts

| Symbiont | Gene | Reference |
| --- | --- | --- |
| *S. ixodetis* | rpoB | Krawczyk et al., 2020 |
| *R. helvetica* | gltA | de Bruin et al., 2015 |
| *B. burgdorferi* s.l. | ospA and flaB | Heylen et al., 2013 |
| *A. phagocytophilum* | ApMSP2 | Courtney et al., 2004 |
| *N. mikurensis* | GroEL | Jahfari et al., 2012 |

**qPCR protocol**All qPCRs were carried out on a LightCycler 480 (Roche Diagnostics Nederland B.V, Almere, the Netherlands) in a final volume of 20 μl with iQ multiplex Powermix, 3 μl of sample, primers with end concentration of 0.2 μM, and probes. Positive plasmid controls and negative water controls were used on every plate tested. Cycling conditions included an initial activation of the iTaq DNA polymerase at 95°C for 5 min, followed by 60 cycles of a 5 s denaturation at 95°C followed by a 35 s annealing‐extension step at 60°C (Ramp rate 2.2°C s−1 and a single point measurement at 60°C) and a final cooling cycle of 37°C for 20 s.

Krawczyk AI, Van Duijvendijk GL, Swart A, Heylen D, Jaarsma RI, Jacobs FH, et al. Effect of rodent density on tick and tick-borne pathogen populations: consequences for infectious disease risk. Parasites & Vectors. 2020;13(1):1-17.

de Bruin A, van Leeuwen AD, Jahfari S, Takken W, Foldvari M, Dremmel L, et al. Vertical transmission of Bartonella schoenbuchensis in Lipoptena cervi. Parasit Vectors. 2015;8:176.

Heylen D, Tijsse E, Fonville M, Matthysen E, Sprong H. Transmission dynamics of Borrelia burgdorferi s.l. in a bird tick community. Environ Microbiol. 2013;15(2):663-73.

Courtney JW, Kostelnik LM, Zeidner NS, Massung RF. Multiplex real-time PCR for detection of anaplasma phagocytophilum and Borrelia burgdorferi. J Clin Microbiol. 2004;42(7):3164-8.

Jahfari S, Fonville M, Hengeveld P, Reusken C, Scholte EJ, Takken W, et al. Prevalence of Neoehrlichia mikurensis in ticks and rodents from North-west Europe. Parasit Vectors. 2012;5:74.
